# Harnessing Enhanced Flame Retardancy in Rigid Polyurethane Composite Foams through Hemp Seed Oil-Derived Natural Fillers

**DOI:** 10.3390/polym16111584

**Published:** 2024-06-03

**Authors:** Mansi Ahir, Chandan Bodhak, Ram K. Gupta

**Affiliations:** 1 Department of Chemistry, Pittsburg State University, 1701 South Broadway Street, Pittsburg, KS 66762, USA; mahir@gus.pittstate.edu; 2 National Institute for Materials Advancement, Pittsburg State University, 1204 Research Road, Pittsburg, KS 66762, USA

**Keywords:** hemp seed oil, bio-filler, rigid polyurethane foam composite, cell morphology, flame retardancy

## Abstract

Over the past few decades, polymer composites have received significant interest and become protagonists due to their enhanced properties and wide range of applications. Herein, we examined the impact of filler and flame retardants in hemp seed oil-based rigid polyurethane foam (RPUF) composites’ performance. Firstly, the hemp seed oil (HSO) was converted to a corresponding epoxy analog, followed by a ring-opening reaction to synthesize hemp bio-polyols. The hemp polyol was then reacted with diisocyanate in the presence of commercial polyols and other foaming components to produce RPUF in a single step. In addition, different fillers like microcrystalline cellulose, alkaline lignin, titanium dioxide, and melamine (as a flame retardant) were used in different wt.% ratios to fabricate composite foam. The mechanical characteristics, thermal degradation behavior, cellular morphology, apparent density, flammability, and closed-cell contents of the generated composite foams were examined. An initial screening of different fillers revealed that microcrystalline cellulose significantly improves the mechanical strength up to 318 kPa. The effect of melamine as a flame retardant in composite foam was also examined, which shows the highest compression strength of 447 kPa. Significantly better anti-flaming qualities than those of neat foam based on HSO have been reflected using 22.15 wt.% of melamine, with the lowest burning time of 4.1 s and weight loss of 1.88 wt.%. All the composite foams showed about 90% closed-cell content. The present work illustrates the assembly of a filler-based polyurethane foam composite with anti-flaming properties from bio-based feedstocks with high-performance applications.

## 1. Introduction

Polyurethanes (PUs) display a pivotal role in the polymer family [1], which belong to a particular class of polymers with unique qualities including low weight, increased durability, and resistance to heat absorption. PU is one of the versatile polymeric materials that finds application in various fields such as elastomers, coatings, paint, adhesives, synthetic skins, foams (rigid or flexible), insulators, and so forth [2,3]. In 1849, the first synthesis of urethane was reported by Wurtz [4], and afterward, in 1937, Otto Bayer synthesized PUs by reacting polyester diol with a diisocyanate [5]. Moreover, it is noteworthy that a relatively small number of PUs were first used as a super-coating on German aircraft during World War II and are now produced at an annual rate of more than 75 thousand tons in the United States [6]. PUs are polymers, typically synthesized by the reaction between the hydroxyl groups (-OH) of a polyol with an isocyanate functional group (-NCO), and the term refers to the resultant urethane linkage [2,4,7]. PU foams (either rigid or flexible) exhibit broad spectrum applications in the building construction, bedding, automotive, packaging, and medical device industries because they offer numerous advantages, which comprise their excellent strength, low thermal conductivity, lightweight nature, and high weight-carrying capacity [8,9]. Indeed, rigid polyurethane foams (RPUFs) account for around 32% of all polyurethane manufacturing and are quite popular because of their low apparent density (10–70 kg/m^3^), excellent compressive strength, low brittleness, less thermal conductivity, and effective thermal insulation [10]. The potent insulating and stable properties of RPUFs become beneficial in tank insulation, refrigeration, and the construction industry [11]. In general, almost 95% of PU foams (PUFs) were made from petroleum sources, of polyols and polyisocyanate, in the presence of a catalyst, which creates the PUR matrix, and simultaneously, isocyanate and water, which produce urea and carbon dioxide, which can act as a blowing agent to create the foam cells.

Over the past few decades, the fluctuating price of crude oil, the depletion of fossil fuel reserves, the low thermal resistance of the product, and increasing environmental concerns have triggered growing interest in the development of bio-renewable feedstocks to replace their petrochemically derived counterparts in the production of some polyurethane (including PUFs) materials [12,13]. In 2011, Tan and co-workers described the feasibility and potential of preparing PU rigid foams from soy-based polyols [14], and later on in 2017, Agrawal et al. conferred to create a brief overview of PU foam derived from renewable sources and demonstrated their perspective on the properties enhancement of PU foam [15]. In this context, in 2019, Carré et al. published a review article that describes different sustainable routes towards the green synthesis of polyhydroxyurethanes (PHUs) material [1]. Thus, there is a trend towards the use of hydroxyl derivatives derived from renewable raw materials to primarily replace petroleum polyol components [16]. In this context, vegetable oils are a viable and sustainable alternative to fossil fuels to produce polyols in the PUF industry because they are affordable, readily available, renewable, and even less expensive than petrochemical raw materials [17,18]. In the manufacture of PUFs ranging from flexible to rigid, several vegetable oils have been used such as castor oil [19,20,21], soybean oil [14,22,23,24,25], palm oil [26], rapeseed oil [27,28], canola oil [29], and tung oil [30]. Indeed, the USA produces about 60% of the world’s soybean oil at a low price with a relatively high degree of unsaturation. In this circumference, Zhang et al. [22] and Sonnenschein et al. [24] individually illustrated the fabrication of soybean oil-based flexible polyurethane foam and its underlying properties. Moreover, vegetable oils become a viable alternative because of the presence of double bonds (odd or even) and hydroxyl groups in the triglyceride chains (castor oil), which can be functionalized or directly utilized to prepare PUFs [17,31].

In continuation, industrial hemp (*Cannabis sativa* L.) is known to be another oilseed crop with excellent potential in the chemical sector, and almost all parts of the hemp plant, including the fibers, seeds, and inflorescence, obtain an industrial application [32]. Hemp seed oil (HSO) is extracted by cold-pressing hemp seeds [33]; despite being a member of the edible oils, it was exempted from cooking due to its low smoke point. HSO is composed of a large content of linoleic acid (55–60%) and linolenic acid (17–35%), with higher iodine values ranging from 140 to 175 g I_2_/100 g, in comparison to soybean oil (128–143 g I_2_/100 g), sunflower oil (110–143 g I_2_/100 g), rapeseed oil (110–126 g I_2_/100 g), and palm oil (44–58 g I_2_/100 g) [33,34]. Recently, Jariwala et al. reported the synthesis of hemp seed oil-based RPUFs and investigated their flame retardancy [35]. Nowadays, industry and academics have focused more on the synthesis and development of PUR composites with natural fillers [36,37]. Polymer composites are essential to many industries, including the building, automotive, aerospace, and packaging sectors [38,39]. Recently, researchers became interested in PUs as a polymer for the fabrication of composites due to their remarkable properties, such as resilience, low-temperature flexibility, durability, and great adhesion [40,41]. An important class of multifunctional PU composite foam materials is stimulated by the combination of foaming and nanoparticles. The incorporation of natural fillers in PUs becomes advantageous because encapsulated fillers can also significantly improve composite PUFs’ properties, indicating higher strength, higher stiffness, and certain special characteristics [42]. Moreover, polyurethane composite foams have also been exploited in the automobile industry as sound absorption material because of their high sound absorption efficiency applications. Concisely, PU composite foams have notable structural properties favorable for a wide range of applications.

In this context, the fabrication of HSO-based RPUF composites using bio-based natural filler is highly prestigious and quite acceptable. Cellulose is a plant-derived ubiquitous natural polysaccharide that is plenteously available in our environment [43,44]. Microcrystalline cellulose has been used in several sectors, such as food, cosmetics, and pharmaceuticals, where it is mostly used as a filler [45]. Septevani et al. [16] reported the impact of cellulose nanocrystals on the thermal conductivity and mechanical properties of RPUFs. In addition to filler, non-halogenated flame retardant (FR) was added to PUR foam to improve its mechanical characteristics and increase its flame retardancy. Melamine is an effective flame retardant for polyurethane foams; however, to improve the mechanical properties of foams, it must be blended with other fillers. In 2020, Członka et al. demonstrated the effect of melamine and silica filler on RPUFs’ characteristics [46]. 

Herein, the main endeavor of our present research is to fabricate an HSO polyol-based rigid polyurethane foam composite and investigate the impact of microcrystalline cellulose filler and non-halogenated melamine flame retardant (FR) on the foams’ properties. The HSO polyol was produced by the epoxidation of HSO followed by oxirane ring-opening using methanol and tetrafluoroboric acid as catalysts. The resultant RPUF composite was subjected to scanning electron microscopy to assess its cell morphology and evaluated for its apparent density, mechanical strength, closed-cell content, and thermal behavior. The flame retardancy of the RPUF composite was also investigated in different loadings of melamine to gain insight into the flame resistance properties of the fabricated composite foam. To the best of our knowledge for the first time, we here investigate the dual impacts of different fillers in the presence of melamine flame retardant on the mechanical strength, thermal stability, cell morphology, and flammability of the RPUF composite. Basically, the individual incorporation of fillers in the foam formulation gives rise to an increase in the mechanical and thermal stability of the foam, while flame retardants generate the anti-flaming ability in the foam characteristics. Here, we successfully demonstrate the combined effect of filler and flame retardant on the properties of the RPUF composite, which could be intriguing research perspectives for industrial application and might open a new avenue in the area of polyurethane research for fellow researchers. 

## 2. Experimental Details

### 2.1. Materials

Cold-pressed raw hemp seed oil (HSO) was happily provided by Midwest Hemp Technology from Kansas-grown hemp grains, extracted through CO_2_ processing. Hydrogen peroxide, tetrafluoroboric acid (HBF_4_), acetic acid, amberlite IR 120H, sodium sulfate, sodium chloride, Lewatit MP 64, toluene, and methanol were purchased from Fisher Scientific, USA. Furthermore, to prepare the RPUF composite, Jeffol SG-522 (sucrose polyol with OH# 522) and Rubinate M isocyanate (methylene diphenyl diisocyanate, MDI) were obtained from Huntsman (The Woodlands, TX, USA). Dibutyltin dilaurate (DBTDL), catalysts NIAX A-1 and 1,4-diazabicyclo[2.2.2]octane (DABCO) T-12 (>95%) were procured from Air Products (Allentown, PA, USA). Also, Tegostab B-8404, used as a silicon surfactant, was acquired from Evonik (Parsippany, NJ, USA). Microcrystalline cellulose 90 μm (Fair Lawn, NJ, USA), alkaline lignin (TRADE TCI MARK), titanium dioxide (UT 84042, Lindon, St. Louis, MO, USA) filler, and melamine as a flame retardant were supplied by Sigma-Aldrich, (St. Louis, MO, USA) along with distilled water (blowing agent) obtained from a local Walmart (Pittsburg, KS, USA). 

### 2.2. Synthesis of Bio-Based Polyol

#### 2.2.1. Synthesis of Epoxidized Hemp Seed Oil (EHSO)

In a 1000 mL three-neck round-bottom flask equipped with a thermometer and a reflux condenser, a stoichiometric amount of cold-pressed filtered HSO and amberlite ion exchange resin (15% by weight of oil) were placed in toluene, and the reaction mixture was mechanically stirred in a water bath for 5 h by maintaining the temperature at 5–10 °C. Once the temperature became steady, acetic acid followed by 30% hydrogen peroxide (0.5:1.5 molar ratio to double bond) was added and stirred continuously for 30 min, and then the temperature was increased to 70 °C and the reaction was continued for another 7 h. After completion of the reaction, indicated by the color change (orange to yellow), the reaction mixture was cooled at room temperature followed by filtration of the amberlite resin, and filtered oil was thoroughly washed with 10% brine solution and then dried over anhydrous sodium sulfate. After that, the extracted oil was concentrated in a rotary evaporator at 55 °C under a vacuum (reduced pressure) to get the epoxidized hemp seed oil.

#### 2.2.2. Synthesis of Hemp Seed Polyols (HSPOs)

**Figure 1:**
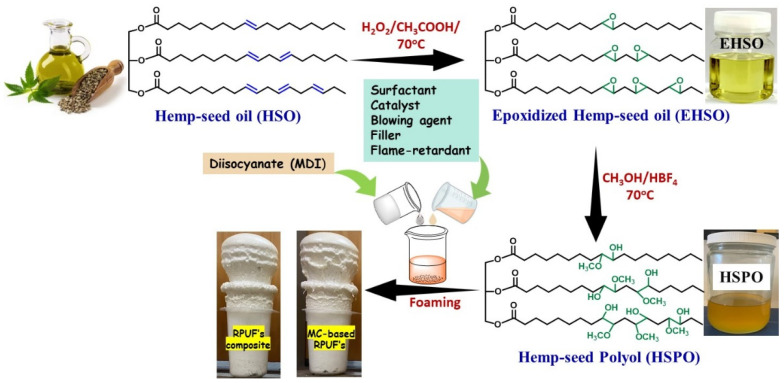
A schematic diagram for the synthesis of HSO-based RPUF composite.

In this reaction, methanol and tetrafluoroboric acid, HBF_4_ (48 wt.% in water), were sequentially added in a 1000 mL four-neck round-bottom flask equipped with a mechanical stirrer, a reflux condenser, a dropping funnel, and a thermometer under a N_2_ atmosphere. Alongside, the temperature of the reaction mixture was maintained at 65–70 °C by an oil bath. Then, EHSO was added dropwise, and the reaction was refluxed for an hour at the same temperature. Methanol and epoxidized HSO were mixed in a mole ratio of 7:1. After that, the reaction mixture was cooled at room temperature followed by the addition of Lewatit MP 64 ion exchange resin to neutralize excess acid. Following that, the resin solution was filtered, and rotary evaporation was carried out under reduced pressure to remove the excess solvents. Finally, the synthesized polyol was characterized by different confirmatory tests such as hydroxyl value and Fourier-transform infrared spectroscopy (FT-IR) to analyze the molecular structure as well as the composition of the substance, and determination of molecular weight was performed via gel permeation chromatography (GPC). The schematic diagram for HSO polyol formation from cold-pressed hemp seed oil is depicted in Figure 1.

### 2.3. Preparation of HSO-Based RPUF Composite

As seen in Figure 1, the RPUFs were prepared in a single pot using a disposable plastic cup mold. Initially, three sets of HPSO-based RPUF composite were prepared using different proportions of fillers like microcrystalline cellulose (MC), alkaline lignin (AL), and titanium dioxide. In these contexts, the F-1 to F-5 samples stand for the formulation of all composite foams containing fillers with the same proportions of other essential constituents (0.18 g of A-1, 0.04 g of T-12, 0.4 g of B8404 surfactant) as shown in Table 1. At first, HSPO, commercial polyol (SG-522), and other additives (including fillers) were mixed thoroughly in a 500 mL disposable plastic cup using a mechanical stirrer at 2000 rpm for 2–3 min to form a homogenous mixture. Then, the pre-measured MDI was added to the blended mixture and stirred vigorously at 2000–2500 rpm for a few seconds, which allowed the composite foam to elevate spontaneously at room temperature. Subsequently, the foam was kept at room temperature for 7 days to ensure complete curing behavior and polymerization. Following the same procedure, a melamine (flame retardant)-based RPUF composite was fabricated as demonstrated in Table 2, where microcrystalline cellulose was incorporated as an effective filler.

### 2.4. Characterization Methods

HSO, EHSO, and HSPO were characterized through different well-established physicochemical methods. The iodine value of HSO was determined by the conventional Hanus method, which measures the average degree of unsaturation per molecule [33]. Additionally, based on ASTM D 4274 [47], the hydroxyl value of HSPO was determined using a phthalic anhydride pyridine (PAP) reagent. Furthermore, tetraethylammonium bromide and glacial acetic acid were used to evaluate the percentage of oxirane oxygen (EOC%) of the EHSO. PerkinElmer Spectrum Two FT-IR spectrophotometers were used to confirm the formation of the EHSO and HSPO from HSO by the detection of specific functional groups. The molecular weights of the HSO, EHSO, and HSPO samples were estimated using a gel permeation chromatography (GPC) instrument (Waters, Milford, MA, USA) equipped with 5 μm phenogel columns in combination with an OMNISEC REVEAL detector and tetrahydrofuran as the eluent solvent (flow rate of 1 mL/min) at 30 °C. The viscosity of the sample was measured at room temperature by the AR 2000 dynamic stress rheometer (TA Instruments, New Castle, DE, USA) fitted with a plate angle of 2° and a cone plate diameter of 25 mm.

Next, to ascertain the physico-mechanical and thermal properties of the HSO-based polyurethane composite foam composite, it was cut into appropriate sizes with specific dimensions. One important factor influencing the physico-mechanical properties and functions of foam is density. The apparent density of the RPUF composite was evaluated according to the ASTM D 1622 [48] method as a ratio of the mass and volume of the specimen foam samples. Scanning electron microscopy (SEM) (Phenom, Rotterdam, The Netherlands) was used for morphological analysis of the foam with a constant acceleration voltage of 10 kV in a chamber under a high vacuum. Composite foam is non-conducting, so to prevent any charging impacts, cube specimens of 0.5 cm^3^ were cut out and pre-coated with a thin layer of gold using a magnetron sputtering instrument (Jefferson Hills, PA, USA).

Furthermore, RPUFs are known to exhibit high closed-cell content, with good insulating properties. In this measurement, an Ultrapycnometer (Ultrafoam 1000, Boynton Beach, FL, USA) was used to measure the closed-cell content (CCC) according to the standard ASTM D 2856 [49] method. The compressive strength test was analyzed by a universal electronic tensile tester named “Q test 2-tensile machine (MTS, Huntsville, AL, USA)” along the direction perpendicular to the foam growth, based on the ASTM D 1621 [50] method. To prepare samples for the compressive strength measurement, foams were cut in a rectangular shape, with the dimension of 50 × 50 × 25 mm^3^ (width × length × height), and a strain rate of 30 mm/min was applied for analysis. The thermal behavior and decomposition of the RPUF composite were analyzed using a TA instrument (TGA Q-500, Delaware, USA) by taking 8–10 milligrams of foam sample under a nitrogen atmosphere with a constant heating rate of 10 °C/min from 30 °C to 700 °C. In addition, the characteristics of the horizontal burning test of the RPUF composite were determined based on the ASTM D 4986-18 [51] standard method to examine the effect of melamine on the fire retardancy of the HSO-based foams. Rectangle-shaped specimens were prepared with dimensions of 150 (length) × 50 (width) × 12.5 (thickness) mm^3^. The weight of the specimens was recorded, they were placed horizontally, and then each foam sample was exposed to flame applied perpendicularly for 10 s. The self-extinguishing time and weight loss were measured after the foam exposition.

## 3. Results and Discussion

### 3.1. Characterization of HSO, EHSO, and HSPO

The iodine value demonstrates the degree of unsaturation in virgin hemp seed oil and its derivatives. The experimental iodine value of hemp seed oil was 142.81 g I_2_/100 g, whereas, in the corresponding epoxide and polyol, the values were 1.30 g I_2_/100 g and 0.85 g I_2_/100 g, respectively, which indicates the successful conversion of the double bond to the prepared polyol. The EHSO was characterized by the epoxy content, EOC%, as 8.01, which confirms the overall epoxidation of HSO. The hydroxyl value shows the number of reactive OH functional groups present in the polyol, and it was one of the most important polyol characteristics to react with isocyanate for the synthesis of polyurethanes. The experimental hydroxyl value and acid value of the synthesized HSPO were 195.11 mg KOH/g and 5.96 mg KOH/g, respectively, which are comparable with the literature [33].

**Figure 2:**
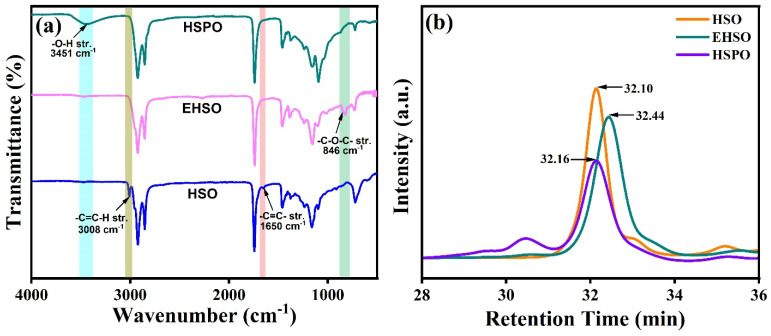
(**a**) FT-IR spectra and (**b**) GPC chromatograms of HSO, EHSO, and HSPO.

The formation of epoxidized oil and polyols from corresponding HSO was confirmed from FT-IR spectra (Figure 2a). In the FT-IR spectra of the HSO, the peaks at 3008 cm^−1^ and 1650 cm^−1^ correspond to the =C-H and -C=C- groups’ stretching vibration present, which is in good correlation with the unsaturation present in vegetable oils [52]. After the epoxidation reaction, the disappearance of =C-H and -C=C- stretching peaks and the appearance of a new peak at 846 cm^−1^, attributed to the oxirane ring vibration peak (C-O-C bending) in the EHSO, confirms the transformation of the epoxidized HSO from the virgin HSO. In the second-step ring-opening reaction, the formation of polyol was confirmed by the disappearance of the epoxy vibration peak at 846 cm^−1^ and the concurrent appearance of a broad hydroxyl (-OH) peak around 3451 cm^−1^. Moreover, the measured viscosity of the HSO polyol was 6.44 Pa·s, whereas for EHSO and HSO, the values were 0.43 Pa·s and 0.04 Pa·s respectively.

The GPC chromatograms of HSO, EHSO, and HSPO are shown in Figure 2b, which demonstrates the progress of the synthesis. In the collected chromatograms, the peaks with 32.10 min and 32.44 min retention times can be attributed to hemp seed oil and epoxidized hemp seed oil, respectively. The chromatogram of HSPO displays a shoulder peak with a 30.7 min retention time, which corresponds to the formation of dimers, and a peak with a retention time of 32.16 min, which determines the increased molecular weight of the polyol. The ring-opening reaction is associated with numerous side reactions, such as oligomerization, which results in dimers, trimers, and oligomers, respectively [53].

### 3.2. Properties of HSO-Based RPUF Composite

#### 3.2.1. Apparent Density and Closed-Cell Content

**Figure 3:**
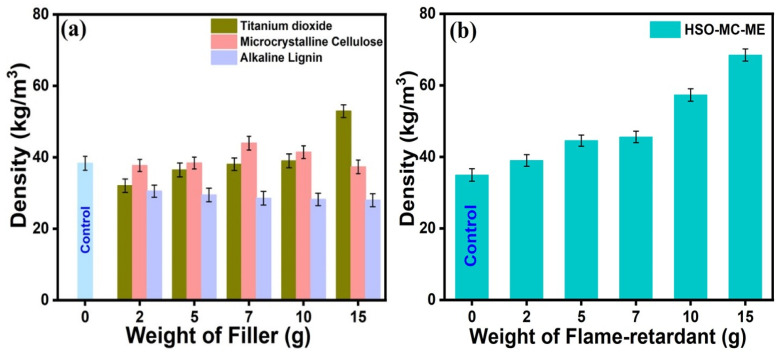
The apparent density of (**a**) filler-based and (**b**) filler-encapsulated flame retardant-based RPUF composite.

The apparent density of RPUF composite is a key parameter affecting the physio-mechanical properties and performances of foam. As shown in Figure 3a, neat RPUFs (control) exhibit a density of 38.4 kg/m^3^, and the density of the corresponding RPUF composite containing different fillers (microcrystalline cellulose, alkaline lignin, and titanium dioxide) have been investigated. In general, the density of RPUF composites falls between 20 and 50 kg/m^3^ without any impact on their cell structures for applications. After that, with a gradual increase in loadings of various fillers, the densities of fabricated HSO-RPUFs obtained are between 30 and 54 kg/m^3^, which is mostly comparable with the literature value of RPUF composites [1,2,54]. The average density of alkaline lignin-based RPUFs was between 28 and 30 kg/m^3^, and this might be due to the aggregation and poor solubility of alkaline lignin in the polyols [55,56], which leads to the weak interaction between alkaline lignin and the polyurethane matrix. In another vein, with increasing the loadings of either microcrystalline cellulose or TiO_2_ fillers, there is a significant trend in the apparent density of RPUF composites in comparison to alkaline lignin, as depicted in Figure 3a. However, the maximum density (53 kg/m^3^) was displayed by the RPUFs with 22.15 wt.% TiO_2_ filler due to the internal structure of the TiO_2_ composites, which significantly influence their mechanical properties. On the other hand, a steady increase in foam density up to 44 kg/m^3^ was observed with 11.72 wt.% microcrystalline cellulose loadings, and further additions resulted in the lowering of the density to a fixed value. In this context, the increment of foam density is attributed to the homogeneous mixing of filler with other foam ingredients, which implies the decrease in average cell size and formation of a uniform cell structure, as indicated by cell morphology (SEM analysis). The void formation in the foam’s cell structures, which significantly affected the foam’s density, may have been the general reason for the lower densities of filler-based RPUF composites. Moreover, RPUF composite containing microcrystalline cellulose became a superior filler over alkaline lignin and TiO_2_ due to its significant apparent density (44 kg/m^3^). Afterward, to check the impact of the flame retardant on the foam’s apparent density along with flame retardancy, we evaluated the density of the microcrystalline cellulose-encapsulated melamine-based RPUF composite. As shown in Figure 3b, notably, with the increase in the melamine loadings, the foam’s density increases gradually and reaches its maxima at 68.49 kg/m^3^, containing 22.15 wt.% melamine, which signifies the impact of the added flame retardant to RPUF composite.

**Figure 4:**
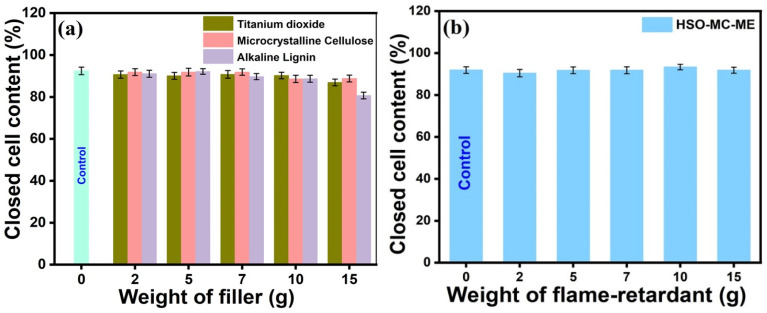
Closed-cell content of (**a**) filler-based and (**b**) filler-encapsulated flame retardant-based RPUF composite.

RPUF is one of the most popular types of thermal insulation, and a high percentage of closed-cell content (CCC) is necessary for it to have excellent insulating performance. The closed-cell content for filler-based RPUFs containing varying filler loadings is depicted in Figure 4a, which demonstrates its comparable closed-cell contents of around 90% in comparison to the control foam specimen. However, the incorporation of melamine flame retardants into the microcrystalline cellulose-based foam caused a slight increase in CCC compared to the control sample, as displayed in Figure 4b. The average range of CCC for all sets of specimen foams falls between 88 and 92%, which reinforces the insulating properties by inhibiting the fast movement of heat and thermal energy between the cells, implying minimal air flow in the void spaces and a limited interconnection of cell structures.

#### 3.2.2. Compressive Strength

**Figure 5:**
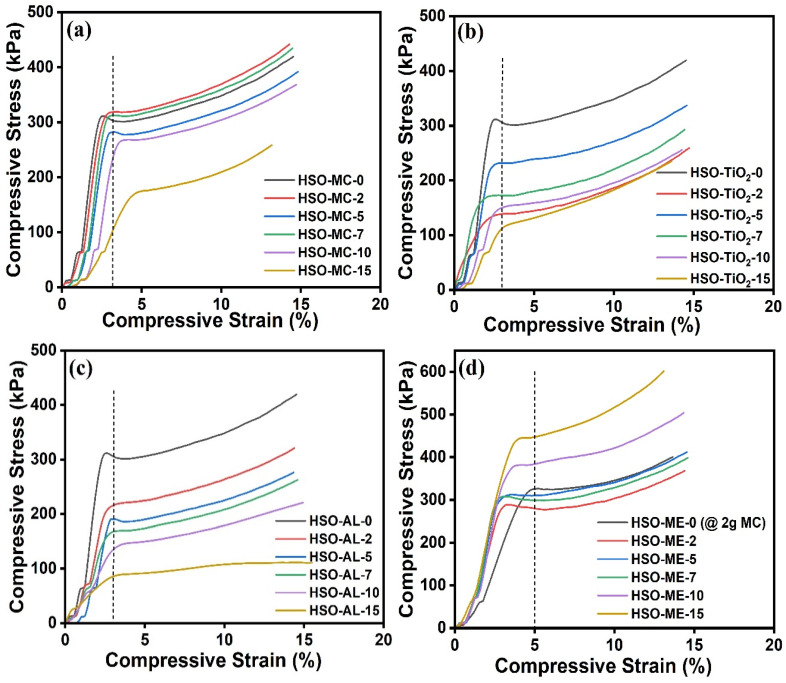
Typical stress-strain curves of the HSO-RPUF composites containing (**a**) MC, (**b**) TiO_2_, (**c**) AL, and (**d**) melamine (MC-encapsulated) under compression, showing linear elastic and plateau regions.

The overall mechanical properties of polyurethanes depend on a few factors, namely apparent density, cellular size, chemical composition, and filler–polymer interaction [57]. The density and cell morphology of foams, as well as the effective dispersion of filler particles into the foam matrix, are major determinants of the specific compressive strength [58]. The compression stress–strain properties and compressive strength of the foams with different fillers and filler-encapsulated flame retardant loadings are presented in Figure 5a–d. A typical stress–strain behavior (compression curves) of RPUF composite exhibits three deformation regions: a linear elastic phase up to small strains, a plateau, and densification [59]. In the elastic regime, the slope of the stress–strain curve characterizes Young’s modulus of foam. It was observed that at 3% deformation, neat foams presented a yield strength of 302 kPa, i.e., before this point, the foam will follow the linear elastic behavior (stress is proportional to deformation) followed by the irreversible plateau region (plastic deformation). The plateau regime is associated with the destruction of the cell wall, where stress does not increase significantly with an increase in strain [59]. As expected, the compressive strength of the RPUF composite increases as the content of different fillers in the polyurethane formulation increases. As depicted in Figure 5a, using microcrystalline cellulose as a green filler, a remarkable compressive strength of the RPUFs was observed. Following the addition of 3.65 wt.% MC (HSO-MC-2), a significant improvement in compressive strength was observed, reaching a maximum (318 kPa). Foam samples HSO-MC-5 (8.66 wt.%) and HSO-MC-7 (11.72 wt.%) exhibit comparable yield strengths of 281 kPa and 312 kPa, respectively, at 3% deformation. After that, no further improvement was observed with increasing MC content, and when using 22.15 wt.% MC (HSO-MC-15), the yield strength value declined to 99 kPa, which closely resembles the apparent density of the composite foam. Additionally, this phenomenon can be associated with more intercalated MC within the polyurethane matrix leading to imposing rigidity in the foam specimen from a mechanical point of view [60]. In general, there exists a strong correlation between the mechanical properties and apparent densities of the foam. The concomitant reduction in apparent density of the alkaline lignin and TiO_2_-embedded foams corroborates with the compression characteristics’ decline. In this context, a gradual decrease in compressive strength was observed for both foam specimens containing alkaline lignin and TiO_2_ filler, and at 3% deformation, the yield strength values of HSO-TiO_2_-15 (22.15 wt.%) and HSO-AL-15 (22.15 wt.%) were found to be lower at 113 kPa and 85 kPa, respectively, compared to neat foam (Figure 5b,c). All these outcomes might be due to the poor dispersion and miscibility of the fillers during the foam formation, which is attributed to the different interactions occurring between the fillers and the polyurethane matrix. On the other hand, microcrystalline cellulose-based RPUF composite containing melamine FR showed an increasing trend in compressive strength and stiffness effect [46]. As indicated in Figure 5d, it was demonstrated that at 5% deformation, the overall range of compressive strength of HSO-based RPUF composite is 280–310 kPa when containing up to 11.72 wt.% melamine loadings (HSO-ME-7), which is mostly comparable with the recorded strength (~320 kPa) of neat foam (containing 2 g MC filler). Indeed, the highest compressive strength was observed at 447 kPa for HSO-ME-15 (22.15 wt.% ME)-formulated foam composite.

#### 3.2.3. Thermogravimetric Analysis

**Figure 6:**
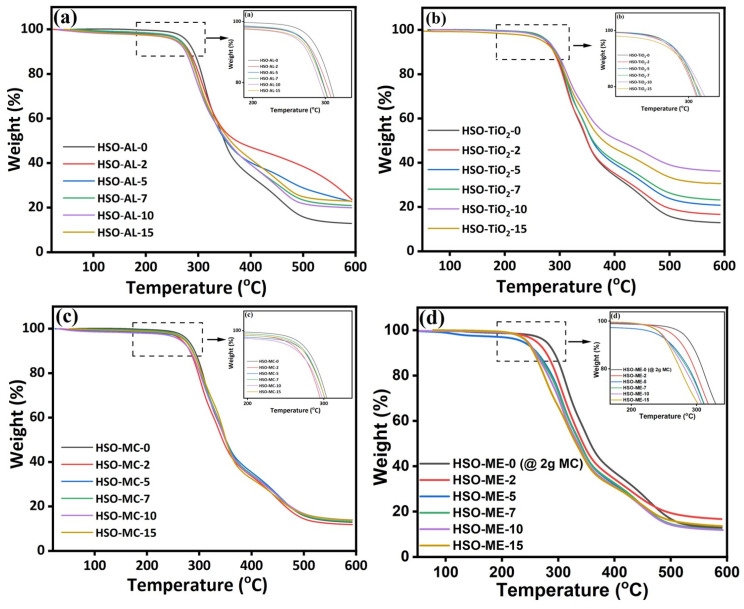
TGA thermograms of HSO-RPUF composites containing (**a**) AL, (**b**) TiO_2_, (**c**) MC, and (**d**) melamine (MC-encapsulated).

Thermogravimetric analysis (TGA) was performed to understand the thermo-oxidative stability of the HSO-RPUF composites under a N_2_ atmosphere, as illustrated in Figure 6, which indicates the TGA curve of the foam sample as a function of temperature and weight loss. The range of thermal degradation is dependent on several factors such as the chemical structure of the components, test conditions, heating rate, etc. In general, the neat PU foam (without any fillers) exhibits two-step degradation stages. The first step, between 200 and 350 °C, is due to the degradation of the hard segments, i.e., the breaking of the urethane linkage [61,62]. The second step is associated with the decomposition of soft segments from 350 °C to 450 °C. In this context, the TGA thermogram strongly supports the degradation pattern of all three RPUF composites and indicates the significant effect of filler loading on the thermal stability of the foam. However, the TGA thermogram of the RPUF composite containing alkaline lignin (AL) exhibits a typical two-step decomposition profile, whereas for both TiO_2_ and microcrystalline cellulose (MC), a three-step degradation pattern was recorded, as shown in Figure 6a–c. Moreover, the TGA thermogram of alkaline lignin-embedded RPUFs displays the first decomposition peak at an early stage (~265 to 280 °C) in comparison to the control foam specimen, which started to decompose in the temperature range of 280 to 320 °C; and also, there was no significant effect of filler loading on the thermal stability of the foam composite (Figure 6a). All the alkaline lignin-incorporated foams with varying concentrations exhibited essentially similar degradation profiles, with an average weight loss of 40–45% for the first decomposition and 20–25% for the second decomposition, respectively.

Indeed, the RPUF composite containing microcrystalline cellulose exhibited first decomposition (degradation of the hard segments) at around 280 to 320 °C, and with the enhancement of filler concentration, there was a gradual decrease in weight loss, which strongly suggests the significant effect of bio-degradable cellulose on the thermal stability of foam composites (Figure 6c). Notably, a diminutive second degradation peak at around 320 to 370 °C was probably due to the thermolysis of cellulose, which produces solid char, vapors, aerosols, and gases such as carbon dioxide. The third decomposition peak at around 375 to 450 °C can be attributed to the breakdown of soft segments and the depolymerization of polyols. On the other hand, although TiO_2_-based RPUF composite displayed a similar three-step degradation pattern (Figure 6b), it is less superior, as early first decomposition (~265 to 280 °C) was observed in comparison to the MC-based RPUF composite. As a consequence, compared to other fillers (TiO_2_ and alkaline lignin), remarkable thermal stability was observed in the MC-grafted composite foam, with less weight residue. It was expected that better thermal stability would be observed once the flame retardant is present in foams. In this context, the TGA thermogram of the microcrystalline cellulose-encapsulated RPUF composite containing melamine flame retardant (MC-ME) represents an analogous degradation profile compared to the cellulosic composite foams, as shown in Figure 6d.

#### 3.2.4. Scanning Electron Microscopy

**Figure 7:**
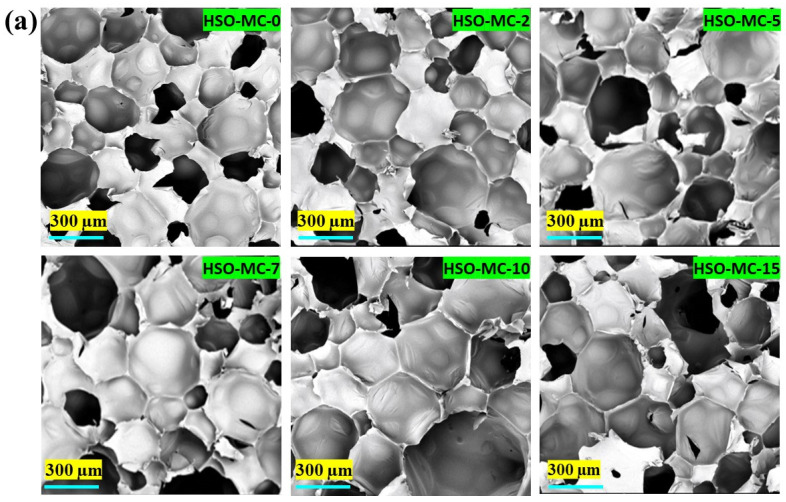

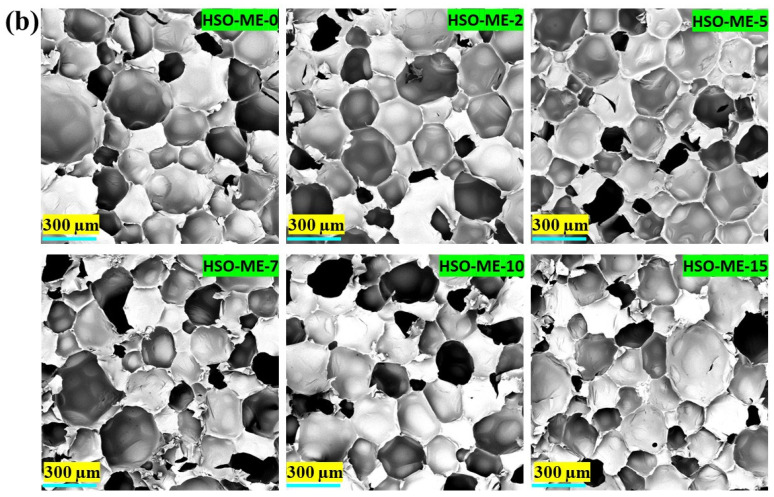
SEM images of HSO-based RPUF composite with (**a**) microcrystalline cellulose filler and (**b**) MC-encapsulated melamine flame retardant.

A study of the microstructure and cell morphology of RPUF composite were investigated by scanning electron microscopy (Figure 7) to get insight into the structural rigidity, apparent density, and mechanical properties of fabricated foam samples. The effect of microcrystalline cellulose or other fillers and flame retardants on the bulk density, cellular structure, as well as the compression strength of foam samples has been documented in the literature [16,63,64]. The average cellular size for neat foam was ~294 μm, with overall uniformly distributed spherical cells, which demonstrates a well-defined closed-cell structure. However, with an increase in the concentration of MC, foams’ cell number increases, leading to a reduction in cell size due to significant interconnection within the cellular structure. The calculated average cell size of MC-encapsulated RPUFs was around 170–225 μm (Figure 7a); this was accomplished by maintaining the relatively uniform spherical cell morphology that affects the compressive strength of the foams. On the other hand, regarding the RPUF composite containing melamine, the average cell size of the control foam sample (encapsulated with 2 g MC) was ~227 μm, whereas with 3.65 wt.% (HSO-ME-2) and 8.66 wt.% (HSO-ME-5), ME loadings displayed a comparable pore size (~220 μm), with a significant increase in cell number in comparison to the control foam sample (Figure 7b). However, at higher loadings of ME (11.72 wt.%, 15.94 wt.%, and 22.15 wt.%), a gradual decrease in average cell size at a range of ~185–200 μm was observed, which corroborates the remarkable increment in compressive strength as well. The aforementioned observation correlates robust particle engagement and substantial interaction between the melamine FR and HSO-based polyurethane matrix without any cell damage in the fabricated RPUF composite.

#### 3.2.5. Horizontal Burning Test

**Figure 8:**
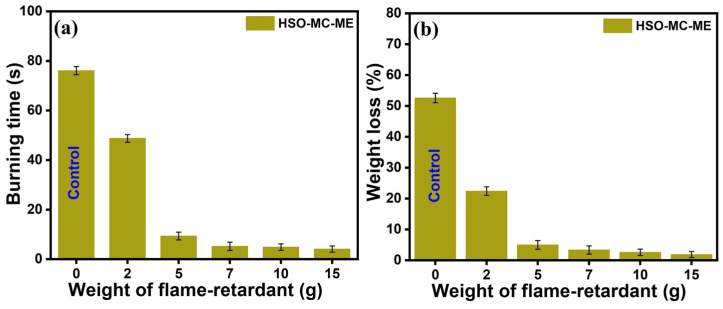
(**a**) Burning time and (**b**) weight loss (%) content of the HSO-RPUFs with varying amounts of melamine.

**Figure 9:**
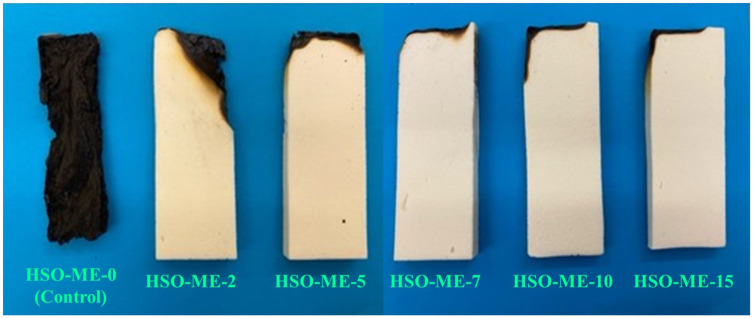
Optical images of horizontal burning tests of HSO-RPUF composites containing melamine flame retardant.

The dual effect of microcrystalline cellulose and melamine flame retardant on the flame retardancy of the hemp seed oil-based RPUF composite was recorded as per the ASTM D 4986-18 standard [51]. The pre-weighted rectangle-shaped foam specimens were placed on the stand vertically, and then each foam was exposed to a direct flame for 10 s inside a fume hood with robust ventilation to remove vapors produced during the ignition process [65]. The samples’ weight loss and burning time were recorded when the flame was quenched, as shown in Figure 8a,b. The burning test was repeated twice by heating the sample from the bottom and top, and the average time was recorded to verify the improved homogeneity and uniform migration of the FR into the foam matrix. The longer the extinguishing time, the greater the weight loss observed in the specimens. Here, the neat foam sample (containing 2 g MC filler) was burned for around 76.1 s with a total weight loss of 52.6 wt.% of its initial weight, which was attributed to the unequal and erratically disrupted cells in the neat PU, which contain trapped air and support prolonged burning [61,66]. It was predicted that the addition of flame retardant would decrease burning time as well as weight loss. In these circumstances, as shown in Figure 8a,b, with the gradual increase in loadings of melamine flame retardant (from 3.65 wt.% to 22.15 wt.%), the corresponding extinguishing time as well as weight loss were reduced, which was accompanied by the impact of melamine FR on the flame retardancy of the RPUF composite. The burning time declined significantly to 4.1 s, with 1.88 wt.% weight loss for the sample containing 15 g ME (22.15 wt.%). This phenomenon of a concomitant reduction in burning time (s) and weight loss (wt.%) of the RPUF composite indicates that an increase in the loadings of melamine significantly enhances the formation of char layers on the surface that could protect the composite foam during combustion, leading to improved flame retardancy. The optical image for the horizontal burning test of HSO-based RPUFs with varying concentrations of melamine FR is shown in Figure 9, which further supports the enhancement of the anti-flaming properties of RPUF composite with embedded flame retardants compared to the neat foam specimen.

Melamine is a nitrogen-based organic flame retardant, that may either release non-flammable gases such as ammonia and nitrogen into the gas phase or undergo a condensation reaction that results in deamination and formation of non-soluble products and condensed derivates such as melam, melem, and melon [46]. Generally, these condensed products create a char layer that obstructs the airway and any further reactive radical groups that could speed up the fire. Additionally, melamine decomposes endothermically, which means it absorbs a lot of heat, lowering the surface temperature of the foam.

## 4. Conclusions

In conclusion, we have demonstrated the fabrication of bio-based rigid polyurethane foam from hemp seed oil using different fillers (microcrystalline cellulose, alkaline lignin, and titanium dioxide) and further exploration towards the impact of melamine as a flame retardant on the mechanical, morphological, thermal and anti-flaming properties of obtained RPUF composites. The bio-based hemp polyol was synthesized by conventional epoxidation followed by the ring-opening of crude hemp seed oil with peracetic acid produced in situ by the reaction of acetic acid and hydrogen peroxide. The rigid polyurethane foams (RPUFs) produced with different fillers like microcrystalline cellulose (MC), alkaline lignin (AL), and titanium dioxide provide a significant impact on the foams’ apparent density, closed-cell content, and compressive strength properties. Notably, MC was shown to be a superior filler over AL and TiO_2_, providing a substantial value in foam density, which is also supported by cell nucleation and changes in cell structures according to the cell morphology of the fabricated RPUFs. The apparent density of composite foam containing 22.15 wt.% melamine FR (HSO-ME-15) reached a maximum of 68.49 kg/m^3^. However, each filler provided a comparable closed-cell content (≥90%), which demonstrates the rigidity level of foam composites. As for the compression strength at 3% deformation, MC filler showed a mostly analogous yield strength in comparison to the neat foam strength of 302 kPa. Indeed, the highest yield strength of 318 kPa was recorded for foam containing 3.65 wt.% MC (HSO-MC-2); this declined to 99 kPa for HSO-MC-15 (22.15 wt.% MC), whereas AL and TiO_2_ displayed inconsistent yield strengths compared to the control foam specimen. On the other hand, the RPUF composite containing 22.15 wt.% melamine flame retardant (HSO-ME-15) exhibited a compressive strength of 447 kPa. Moreover, the TGA thermogram further elucidates the thermal behavior of the RPUF composite. Additionally, flame retardancy was significantly ascribed to the incorporation of melamine as a non-halogenated flame retardant. In this context, the burning test experiment revealed that an increase in melamine loading gave rise to subsequent lowering of extinguishing time along with weight loss of the sample, and a maximum of 1.88 wt.% of weight loss was observed in 4.1 s (HSO-ME-15) compared to the control sample (52.6 wt.% and 76.1 s). In a nutshell, the fabrication of natural filler-encapsulated rigid polyurethane foam composites containing non-halogenated flame retardant from bio-based hemp seed oil in a sustainable manner became appealing and intrigued the present researchers to use them in several industrial and technical applications, owing to their enriched mechanical, thermal, and good fire-resistance properties, which might be a new avenue for future perspectives.

## Figures and Tables

**Table 1:** Formulation for the preparation of RPUFs with filler variations.

Ingredient	Control	F-1	F-2	F-3	F-4	F-5
Hemp polyol	10	10	10	10	10	10
SG-522 polyol	10	10	10	10	10	10
A-1	0.18	0.18	0.18	0.18	0.18	0.18
Water	0.8	0.8	0.8	0.8	0.8	0.8
T-12	0.04	0.04	0.04	0.04	0.04	0.04
B8404	0.4	0.4	0.4	0.4	0.4	0.4
Diisocyanate (MDI)	31.29	31.29	31.29	31.29	31.29	31.29
AL ^a^ or TiO_2_ or MC ^b^ (g)	0	2	5	7	10	15
AL ^a^ or TiO_2_ or MC ^b^ (%)	0	3.65	8.66	11.72	15.94	22.15

^a^ AL = alkaline lignin, ^b^ MC = microcrystalline cellulose.

**Table 2:** Formulation for the preparation of RPUF composite.

Ingredients	Control	F-1	F-2	F-3	F-4	F-5
Hemp polyol	10	10	10	10	10	10
SG-522 polyol	10	10	10	10	10	10
A-1	0.18	0.18	0.18	0.18	0.18	0.18
Water	0.8	0.8	0.8	0.8	0.8	0.8
T-12	0.04	0.04	0.04	0.04	0.04	0.04
B8404	0.4	0.4	0.4	0.4	0.4	0.4
Diisocyanate (MDI)	29.29	29.29	29.29	29.29	29.29	29.29
MC (g)	2	2	2	2	2	2
Melamine (g)	0	2	5	7	10	15
Melamine (%)	0	3.65	8.66	11.72	15.94	22.15

## Data Availability

Data are contained within the article. Further inquiries can be available on request from the corresponding author.
